# Complete Genome Sequence of *Streptomyces* Phage Shady

**DOI:** 10.1128/MRA.01336-20

**Published:** 2021-01-07

**Authors:** Carlos Ortega, Isla Hernandez, James Clark, Mei Liu, Ben Burrowes

**Affiliations:** a Department of Biochemistry and Biophysics, Texas A&M University, College Station, Texas, USA; b Center for Phage Technology, Texas A&M University, College Station, Texas, USA; Queens College

## Abstract

*Streptomyces* spp. are saprophytic soil bacteria that produce secondary metabolites with therapeutic potential. This announcement describes the isolation and genome annotation of *Streptomyces* sp. strain Mg1 siphophage Shady. Learning more about Shady’s novel 45-kb genome, containing 76 predicted protein-coding genes, could be industrially advantageous when using streptomycetes for their products.

## ANNOUNCEMENT

*Streptomyces* spp. are common, soilborne Gram-positive bacteria lacking motility organelles. Many streptomycetes, such as *Streptomyces* sp. strain Mg1, produce secondary metabolites that degrade competitor colonies (1, 2). These metabolites account for over 70% of naturally available antibiotics. Studying bacteriophages that can spoil *Streptomyces* cultures could be beneficial to the drug industry (3–5). This report details the isolation and genome annotation of Shady, a *Streptomyces* sp. Mg1 siphophage.

Shady was isolated from soil collected in Houston, Texas, in August 2019 and propagated on *Streptomyces* sp. Mg1 (provided by Paul Straight, Texas A&M University) by plaque purification in nutrient broth or agar with 10 mM MgCl_2_, 8 mM Ca(NO_3_)_2_, and 0.5% glucose (6). Samples were negatively stained with 2% (wt/vol) uranyl acetate and imaged at the Texas A&M Microscopy and Imaging Center using transmission electron microscopy (data not shown). Shady DNA was purified using a modified Wizard DNA clean-up kit (Promega) protocol as described elsewhere (7). iSeq libraries were prepared with 300-bp inserts using a TruSeq Nano kit and sequenced using V2 500-cycle chemistry on an Illumina MiSeq instrument with paired-end 250-bp reads. FastQC was used to visualize and quality control the 325,742 reads (www.bioinformatics.babraham.ac.uk/projects/fastqc) which were then trimmed with FastX Toolkit 0.0.14 (http://hannonlab.cshl.edu/fastx_toolkit/download.html), and one contig was assembled at 107.3-fold coverage using SPAdes v3.5.0 (8). The genome was closed using PCR and Sanger sequencing, (forward primer, 5′-GAGGAATCCATCTAGCGAAGTC-3′; reverse primer, 5′-ATAATGCGGATCTGTTCGGG-3′). PhageTerm was used to predict the genomic termini (9). MetaGeneAnnotator v1.0 (10) and GLIMMER v3 (11) were used to predict protein-coding genes, and ARAGORN v2.36 (12) was used to detect tRNA genes. To predict gene function, conserved domain searches with InterProScan v5.33 (13), transmembrane domain searches with TMHMM v2.0 (14), and similarity searches with BLAST v2.9.0 (15) versus the NCBI nonredundant (nr), Swiss-Prot, and TrEMBL databases (16) were utilized at the default settings (accessed 24 March 2020). Similarity calculations of the genome-wide DNA sequence were carried out using progressiveMauve v2.4 (17). Tools were accessed via the Center for Phage Technology Web Galaxy interface online at https://cpt.tamu.edu/galaxy-pub, using Web Apollo for annotations (18–20). Default parameters were used for all software unless otherwise specified.

The 45,128-bp double-stranded DNA (dsDNA) genome of Shady has a GC content of 62.3%, which is significantly less than the 72.2% of its host (1). Genomic analysis predicted 76 protein-coding and 8 tRNA genes, yielding a coding density of 89.3%. PhageTerm could not detect genomic termini.

Of the 76 identified protein-coding genes, 33 were assigned putative functions. Various putative tail protein genes were found with high similarity to *Mycobacterium* phages Bxb1 and L5. They include two tape measure protein chaperone genes embedded in the same exon with a slippery sequence causing a frameshift resulting in two different products. Putative DNA primase and DNA polymerase I genes were identified, indicating that Shady might form at least some of its own DNA replication machinery. Furthermore, a putative integrase gene very closely related to lambdoid phage phiC31 was found, suggesting that Shady is a temperate phage (4). Transmission electron microscopy showed the virion to be a siphovirus, and comparative genomics revealed Shady to have no close relatives. Shady is therefore a novel *Streptomyces* siphophage.

### Data availability.

The genome sequence of Shady is available in GenBank (accession number MT701596.1). The associated BioProject, SRA, and BioSample accession numbers are PRJNA222858, SRR11558351, and SAMN14609630, respectively.
